# Associations between tobacco smoking and mortality: a sex-stratified cohort analysis

**DOI:** 10.1093/eurpub/ckaf194

**Published:** 2025-10-24

**Authors:** Alexandre Vallée

**Affiliations:** Department of Epidemiology and Public Health, Foch Hospital, Suresnes, France

## Abstract

To investigate the associations between tobacco smoking and mortality, focusing on all-cause, cardiovascular, and cancer mortality, with analyses stratified by sex. A total of 333 559 participants were included. Smoking status was categorized as current, past, or never. Cox proportional hazards regression models estimated hazard ratios (HRs) and 95% confidence intervals (CIs) for mortality outcomes, adjusted for potential confounders. During a median follow-up of 11.8 years, 20 381 deaths occurred, including 4024 cardiovascular deaths. Current smokers had substantially increased risks of all-cause mortality (HR 2.37 [2.25–2.50] in males; HR 2.65 [2.47–2.84] in females), cardiovascular mortality (HR 2.58 [2.31–2.87] in males; HR 3.79 [3.17–4.54] in females), and cancer mortality (HR 2.47 [2.30–2.66] in males; HR 2.46 [2.25–2.69] in females) compared with never-smokers. Past smokers also exhibited elevated risks, and a clear dose–response relationship was observed with increasing smoking intensity and pack-years. Overall survival was higher in females, but the relative risks associated with smoking were largely comparable across sexes. Tobacco smoking is strongly associated with increased mortality risk, showing a clear dose–response relationship and long-term adverse effects even after cessation. The detrimental impact of smoking was broadly similar in males and females, with only minor differences. These findings reinforce the urgent need for universal prevention and cessation strategies to reduce the burden of smoking-related disease.

## Introduction

Tobacco smoking is the foremost preventable cause of premature mortality, contributing to over six million deaths annually across the globe [1]. Smoking-related deaths include those from cardiovascular disease (CVD), cancer, and other chronic conditions, with significant variations based on smoking intensity, duration, and cessation history [2].

Epidemiological studies have consistently demonstrated that smoking substantially increases the risk of all-cause mortality [3, 4]. Among smoking-related deaths, cardiovascular mortality and cancer mortality account for a large proportion [5]. Even low levels of tobacco exposure have been associated with a significant increase in mortality risk, challenging the misconception that occasional or light smoking is relatively harmless [6]. Individuals who smoke even a single cigarette per day face a markedly higher risk of death compared to never-smokers [7]. The mortality burden of smoking is not limited to active smokers; exposure to secondhand smoke has also been linked to increased mortality risks, particularly from CVD and cancer [8].

The toxic composition of cigarette smoke plays a central role in the development of fatal diseases. Cigarette smoke contains numerous chemical compounds, including at least 72 known carcinogens, as well as numerous toxicants that contribute to cardiovascular and respiratory diseases [2]. While the association between smoking and mortality is well established, the specific biological mechanisms through which different chemical components drive disease progression remain an area of ongoing research. 1,3-butadiene has been linked to cancer risk, while cyanide, arsenic, and cresols have been associated with cardiovascular toxicity [2].

Despite extensive global efforts to reduce smoking prevalence, a substantial proportion of the population continues to smoke, including occasional and light smokers who may underestimate their risk [6]. Understanding the impact of smoking on mortality and the differential risks associated with CVD and cancer deaths is crucial for guiding public health interventions and regulatory policies. This study aimed to examine the associations between tobacco smoking and all-cause, cardiovascular, and cancer mortality in a large population cohort. This study assessed both smoking status and cumulative exposure to characterize dose–response relationships and further conducted sex-stratified analyses to explore whether associations differed between males and females.

## Methods

### UK biobank population

The UK Biobank stands as a forward-looking cohort initiative aimed at investigating, preventing, diagnosing, and treating chronic diseases, notably CVD in adults. This extensive study encompassed 502 478 individuals from across 22 cities in the UK, all registered with the UK National Health Service. Participants were aged between 40 and 69 years when they joined UK Biobank between 2006 and 2010. The collection of biological samples including blood, urine, and saliva was integral to the study [9]. Data include socioeconomic variables, behavioral and lifestyle information, a comprehensive mental health assessment, as well as clinical diagnoses and treatments. The protocol that guided this extensive collection and analysis of data is thoroughly documented in the scientific literature [10].

### Ethical considerations

All participants in the study provided their informed consent electronically. The UK Biobank obtained ethical approval from the North-West Multi-center Research Ethics Committee, which extends its jurisdiction across the entire UK. The study adhered to the principles outlined in the Declaration of Helsinki and received approval from the Northwest—Haydock Research Ethics Committee. The specific protocol code for this approval was 21/NW/0157, and the date of approval was 21 June 2021. For more detailed information: https://www.ukbiobank.ac.uk/learn-more-about-uk-biobank/about-us/ethics

### Study population

**Figure 1.**
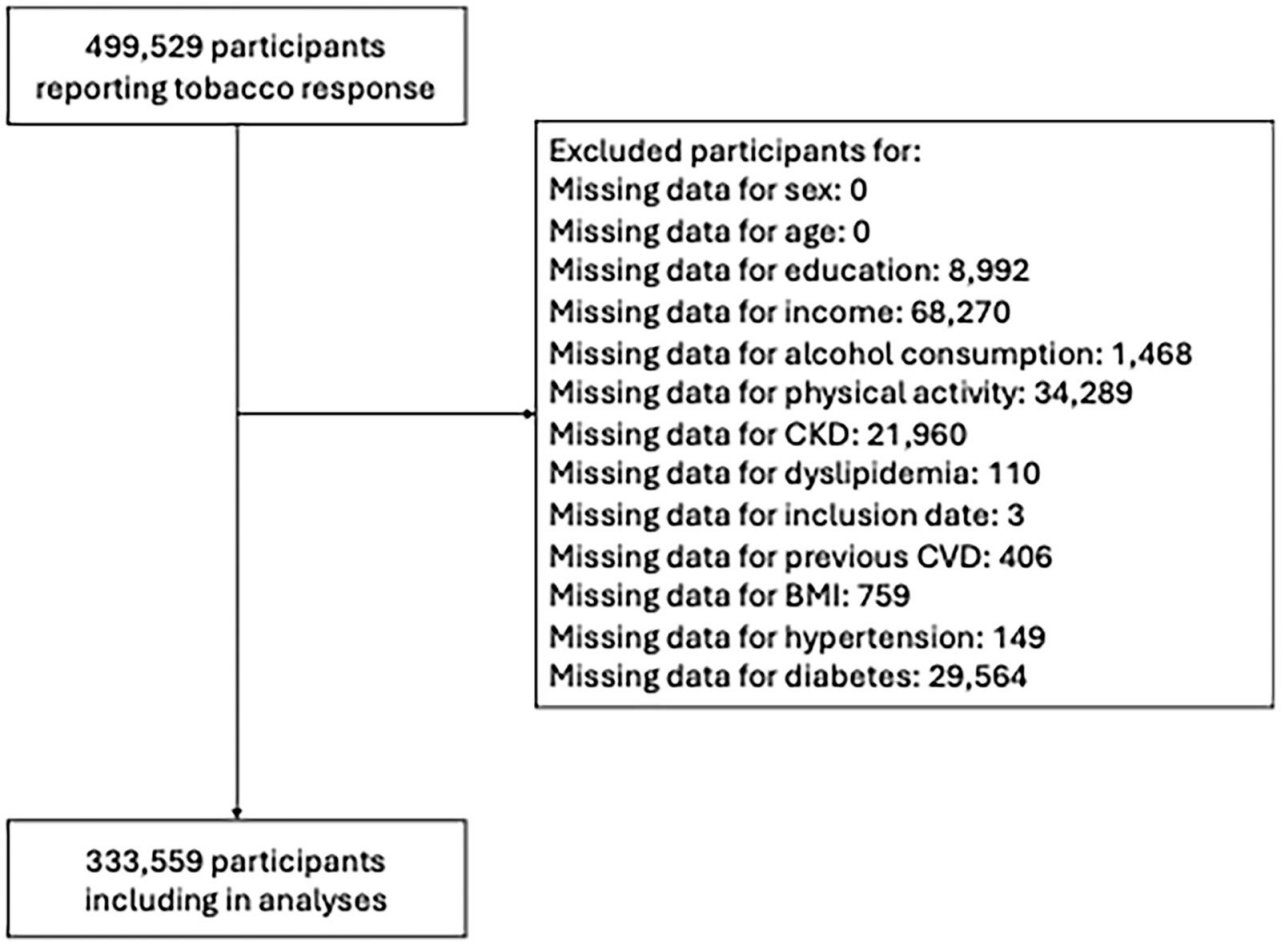
Flowchart.

499 529 participants of the UK Biobank who responded to the questions of tobacco smoking use were included. Of them, 165 970 were excluded for missing data. Therefore, the study analyzed 333 559 participants (Fig. 1).

### Ascertainment of mortality

Participant monitoring for mortality in the UK Biobank study commenced from the point of their inclusion. This follow-up was systematically concluded on 19 December 2020, for all participants. Data regarding the causes of death were sourced from the National Health Service Information Center. For those interested in the specifics of how this data was linked and processed, comprehensive details are accessible online at the following link: http://biobank.ctsu. ox.ac.uk/crystal/refer.cgi? id=115559

Causes of cardiovascular mortality were defined with main cause of death corresponding to ICD10 codes: I00–I78; G951, H341, H342, O10, and S066 [11].

Causes of cancer mortality were defined with main cause of death corresponding to ICD10 codes: C00–C96, and D00–D48 [11].

### Tobacco smoking

Participants were categorized by self-report, as “current,” “past,” “never” smokers. Current tobacco smokers were defined as participants who responded “yes, on most or all days” or “yes, only occasionally” to the question: “Do you smoke tobacco now?.” Smoking pack-years were calculated for individuals who have ever smoked. Smoking pack-years were calculated as the average number of packs smoked per day multiplied by the total number of years of smoking in lifetime. The general definition of a pack-year is the number of cigarettes smoked per day, divided by 20, multiplied by the number of years of smoking. In the UK Biobank, the number of years of smoking is calculated by subtracting the age of starting smoking from the age smoking was stopped (or age at inclusion for current smokers), using the equation:


Pack-years = Number of cigarettes per day/20 × (age stopped smoking−age started smoking)

For current smokers, the participants had to respond to: “About how many cigarettes do you smoke on average per day?”; and for past smokers: “About how many cigarettes did you smoke on average per day?.” Participants who responded “never smoked” were allocated zero for both smoking pack-years and cigarettes per day [12]. Smoke pack years users were defined as: never (zero pack years), less than 15 smoke pack years, 15–24 smoke pack years, and more than 25 pack years [13, 14].

Cigarettes consumption was defined as: never (zero cigarettes per day), less than 20 cigarettes per day, between 20 and 39 cigarettes and more than 40 cigarettes [15].

### Covariates

Systolic and diastolic blood pressure (SBP/DBP) were measured twice at inclusion using an automated device (Omron 705 IT), or manually if needed. Hypertension was defined as SBP ≥140 mmHg and/or DBP ≥90 mmHg, use of antihypertensive medication, or physician diagnosis, in line with ESC guidelines [16].

Diabetes was defined by anti-diabetic treatment, medical diagnosis, or fasting glucose ≥7 mmol/l [17]. Dyslipidemia was defined by total cholesterol ≥6.61 mmol/l, LDL ≥4.1 mmol/l, triglycerides >1.7 mmol/l, or statin use. Medication use was self-reported.

Cardiovascular disease included self-reported history of heart attack, angina, or stroke diagnosed by a physician. BMI was calculated as weight/height [2] and categorized as low (<25 kg/m^2^), overweight (25–30), or obese (>30). Detailed information on biological parameters can be found in the UK Biobank protocol [18].

Education was categorized as high (university), intermediate (A/AS/O levels or vocational), or low (none). Income was high (>£52 000/year), moderate (£18 000–52 000), or low (<£18 000).

Estimated glomerular filtration rate (eGFR) was calculated based on the Chronic Kidney eGFR was calculated using the CKD-EPI formula, with CKD defined as eGFR <60 ml/min/1.73 m^2^. e‐GFR <60 ml/min/1.73 m^2^ defined chronic kidney disease (CKD).

Alcohol use was self-reported as current, past, or never. Weekly alcohol intake (in UK units) was derived by summing reported units from various drinks; monthly reports were converted to weekly, then daily. Non-drinkers were assigned zero consumption [19].

Physical activity was assessed via a revised IPAQ completed on a tablet. MET-hours/week were computed following Bradbury *et al.* and categorized as low (<10), moderate (10–49.9), or high (≥50) [20].

### Statistical analysis

Characteristics of the study population were described as the means with standard deviation (SD) for continuous variables. Categorical variables were described as numbers and proportions. Comparisons between groups were performed using Student’s t-test for continuous variables, an ANOVA analysis was performed to assess the difference between several groups. Pearson’s *χ*^2^ test was performed for categorical variables. Statistical analyses were stratified on sex due to tobacco consumption differences between males and females [21, 22].

Separate Cox proportional hazards regression models were fitted for males and females to estimate sex-specific hazard ratios (HRs) and 95% confidence intervals (CIs) for mortality outcomes. Each model was adjusted for the same set of confounders: age, education, income, alcohol consumption, dyslipidemia, BMI, physical activity, CKD, previous CVD, hypertension and diabetes.

In addition, an interaction analysis has been conducted to test whether the association between tobacco use, and mortality differed by sex, by including a multiplicative interaction term in the combined model. Kaplan Meier analyses censored at 10 years follow-up were performed and compared by log-rank test. Follow-up time for each participant was calculated as the difference between the examination date in the UK Biobank and the last known date alive (19 December 2020) or censored from the linked mortality-life.

To address the interpretability issues associated with interaction terms, we created a six-category combined variable representing sex and smoking status. Cox models were refitted using this variable (with Male never smokers as reference), adjusting for the same covariates.

“Never users” of tobacco use was considered as the referent group in the analyses. Statistics were performed using SAS software (version 9.4; SAS Institute, Carry, NC). A *P* values < .05 was considered statistically significant.

## Results

Among 333 559 participants (51.1% females with mean [SD] age of 55.67 [7.99] years; 48.9% males with mean [SD] age of 56.53 [8.17] years) during an overall median of 11.83 years (IQR, 11.09–12.53 years) of follow-up, 20 381 total deaths occurred, including 4024 deaths from CVD and 10 869 from cancer (Table 1).

**Table 1.** Characteristics of the study population according to sex

	Males *N* = 162 975		Females *N* = 170 584		
	*N*/mean	%/SD	*N*/mean	%/SD	*P* value
**Age**	56.53	8.17	55.67	7.99	<.001
**Tobacco status**					<.001
current	19 400	11.90%	15 022	8.81%	
past	63 001	38.66%	54 652	32.04%	
never	80 574	49.44%	100 910	59.16%	
**Alcohol status**					<.001
current	153 679	94.30%	156 608	91.81%	
past	5470	3.36%	5753	3.37%	
never	3826	2.35%	8223	4.82%	
**Alcohol consumption (g/day)**	2.96	3.00	1.57	1.93	<.001
**Education**					<.001
high	60 438	37.08%	60 864	35.68%	
moderate	70 466	43.24%	79 052	46.34%	
low	32 071	19.68%	30 668	17.98%	
**Income**					<.001
high	47 215	30.07%	41 421	24.92%	
moderate	80 439	51.24%	86 589	52.08%	
low	29 345	18.69%	38 237	23.00%	
**Previous CVD**	13 020	7.99%	5049	2.96%	<.001
**Hypertension**	95 309	58.48%	63 044	36.96%	<.001
**Diabetes**	13 882	8.52%	8781	5.15%	<.001
**Dyslipidaemia**	105 675	64.84%	83 757	49.10%	<.001
**Cigarettes consumption classification**					<.001
>40 cigarettes per day	4841	3.57%	1258	0.85%	
20–39 cigarettes per day	27 677	20.42%	18 342	12.46%	
<20 cigarettes per day	22 424	16.54%	26 591	18.07%	
never	80 629	59.47%	100 971	68.61%	
**Smoke pack years classification**					<.001
>25 smoke pack years	23 167	17.06%	13 651	9.35%	
15–24 smoke pack years	12 504	9.21%	10 621	7.27%	
<15 smoke pack years	19 250	14.17%	20 657	14.14%	
never	80 884	59.56%	101 142	69.24%	
**Physical activity**					<.001
high	37 596	23.07%	32 823	19.24%	
moderate	82 989	50.92%	90 604	53.11%	
low	42 390	26.01%	47 157	27.64%	
**CKD**	7608	4.67%	1147	0.67%	<.001
**BMI**	27.78	4.19	26.92	5.11	<.001
**BMI level**					<.001
high	40 583	24.90%	38 305	22.46%	
overweight	81 080	49.75%	62 287	36.51%	
low	41 312	25.35%	69 992	41.03%	
**Overall deaths**	12 970	7.96%	7411	4.34%	<.001
**CVD deaths**	3031	1.98%	993	0.60%	<.001
**Cancer deaths**	6294	4.03%	4575	2.73%	<.001

Males were associated with higher risk of all-cause mortality with HR = 1.48 (95% CI, 1.43–1.53) and a significant interaction between sex and tobacco smoking (*P* = .039). Similar results were observed with CVD mortality [males, HR= 2.14 (95% CI, 1.97–2.31)] and a significant interaction between sex and tobacco (*P* = .020). Similar results were observed with cancer mortality [males, HR= 1.27 (95% CI, 1.21–1.33)] but without significant interaction between sex and tobacco (*P* = .224).

**Figure 2.**
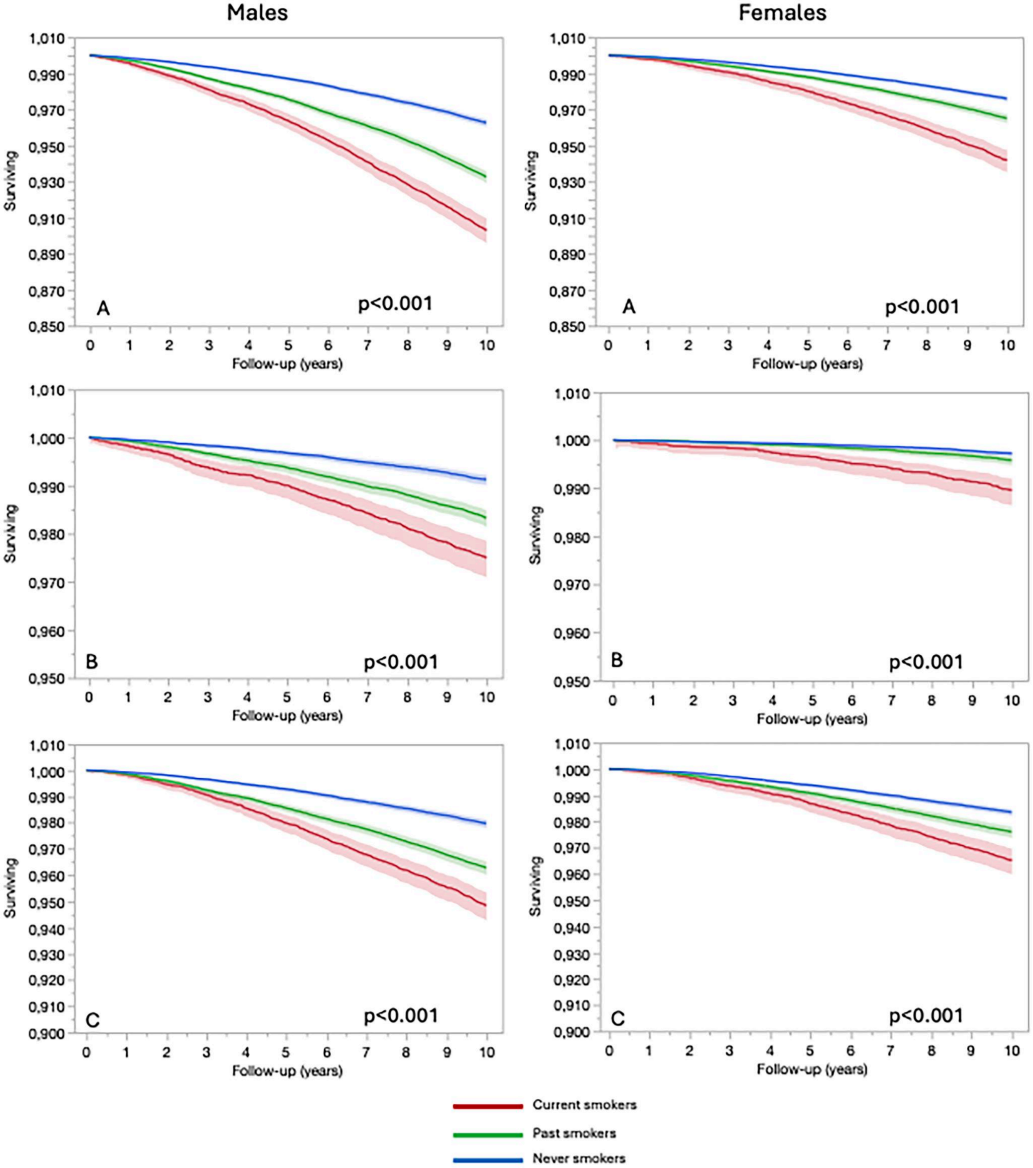
Kaplan Meier for overall mortality (A), CVD mortality (B), and cancer mortality (C) in males and females according to tobacco smoking status.

In males, after full adjustment, the hazard ratios (HRs) were 2.37 (95% CI, 2.25–2.50) for all-cause mortality, 2.58 (95% CI, 1.24–1.36) for CVD mortality, and 2.47 (95% CI, 2.30–2.66) for cancer mortality among current tobacco users compared to never users (Table 2). In females, after full adjustment, the HRs were 2.65 (95% CI, 2.47–2.84) for all-cause mortality, 3.79 (95% CI, 3.17–4.54) for CVD mortality, and 2.46 (95% CI, 2.46–2.69) for cancer mortality among current tobacco users compared to never users (Table 2, Fig. 2).

**Table 2.** Sex-stratified cox regression models for all-cause, CVD, and cancer mortality according to tobacco smoking

Tobacco smoking status			
Mortality	Tobacco	Males	Females
Overall	Current	2.37 [2.25–2.50]	2.65 [2.47–2.84]
	Past	1.28 [1.22–1.33]	1.26 [1.20–1.32]
	Never	Ref.	Ref.
CVD	Current	2.58 [2.31–2.87]	3.79 [3.17–4.54]
	Past	1.24 [1.13–1.36]	1.26 [1.08–1.46]
	Never	Ref.	Ref.
Cancer	Current	2.47 [2.30–2.66]	2.46 [2.25–2.69]
	Past	1.30 [1.23–1.38]	1.30 [1.22–1.39]
	Never	Ref.	Ref.

Smoke pack years classification			
Mortality	Tobacco	Males	Females
Overall	>25 smoke pack years	2.14 [2.04–2.25]	2.41 [2.25–2.57]
	15–24 smoke pack years	1.36 [1.27–1.46]	1.58 [1.45–1.73]
	<15 smoke pack years	1.11 [1.04–1.19]	1.20 [1.11–1.30]
	never	Ref.	Ref.
CVD	>25 smoke pack years	1.99 [1.80–2.21]	2.80 [2.35–3.33]
	15–24 smoke pack years	1.40 [1.22–1.362	1.79 [1.41–2.28]
	<15 smoke pack years	1.14 [0.99–1.31]	1.25 [0.99–1.56]
	never	Ref.	Ref.
Cancer	>25 smoke pack years	2.36 [2.20–2.53]	2.43 [2.23–2.65]
	15–24 smoke pack years	1.37 [1.24–1.51]	1.62 [1.45–1.81]
	<15 smoke pack years	1.16 [1.06–1.27]	1.23 [1.12–1.36]
	never	Ref.	Ref.

Cigarettes per day classification			
Mortality	Tobacco	Males	Females
Overall	>40 cigarettes per day	1.96 [1.81–2.13]	2.43 [2.05–2.89]
	20–39 cigarettes per day	1.70 [1.62–1.79]	1.82 [1.71–1.85]
	<20 cigarettes per day	1.44 [1.37–1.53]	1.57 [1.47–1.67]
	never	Ref.	Ref.
CVD	>40 cigarettes per day	1.63 [1.37–1.94]	2.87 [1.88–4.40]
	20–39 cigarettes per day	1.64 [1.48–1.83]	2.03 [1.69–2.43]
	<20 cigarettes per day	1.48 [1.32–1.66]	1.87 [1.58–2.22]
	never	Ref.	Ref.
Cancer	>40 cigarettes per day	2.25 [2.00–2.51]	2.19 [1.72–2.79]
	20–39 cigarettes per day	1.79 [1.67–1.92]	1.90 [1.74–2.07]
	<20 cigarettes per day	1.50 [1.38–1.61]	1.54 [1.42–1.67]
	never	Ref.	Ref.

In males, more than 25 pack years smoking, after full adjustment, was associated with all-cause mortality (HR, 2.14; 95% CI, 2.04–2.25), CVD mortality (HR, 1.99; 95 CI, 1.80–2.21), and cancer mortality (HR, 2.26; 95% CI, 2.20–2.53) compared to never users. In females, more than 25 pack years smoking, after full adjustment, was associated with all-cause mortality (HR, 2.41; 95% CI, 2.25–2.57), CVD mortality (HR, 2.80; 95 CI, 2.35–3.33), and cancer mortality (HR, 2.43; 95% CI, 2.23–2.65) compared to never users (Table 2, Fig. S1).

In males, more than 40 cigarettes per day, after full adjustment, was associated with all-cause mortality (HR, 1.96; 95% CI, 1.81–2.13), CVD mortality (HR, 1.63; 95 CI, 1.37–1.94), and cancer mortality (HR, 2.25; 95% CI, 2.00–2.51) compared to never users. In females, more than 40 cigarettes per day, after full adjustment, was associated with all-cause mortality (HR, 2.43; 95% CI, 2.05–2.89), CVD mortality (HR, 2.87; 95 CI, 1.88–4.40), and cancer mortality (HR, 2.19; 95% CI, 1.72–2.79) compared to never users (Table 2, Fig. S1). Significant associations were observed among past tobacco smokers for all-cause mortality, CVD and cancer mortality (Table 2).

To facilitate the interpretation of the significant interaction between sex and smoking status, we created a combined six-level variable (sex × smoking status) and refitted the Cox models accordingly. Table S1 presents the adjusted HRs for all-cause, cardiovascular (CVD), and cancer mortality, using male never-smokers as the reference group. Current male smokers had the highest mortality risks for all outcomes: HR 2.42 (95% CI: 2.31–2.55) for all-cause mortality, HR 2.62 (95% CI: 2.37–2.91) for CVD mortality, and HR 2.56 (95% CI: 2.38–2.74) for cancer mortality. Compared to male never-smokers, current female smokers also had increased risks, especially for cancer mortality (HR 2.00 [1.83–2.18]), but their relative risks were lower than those of their male counterparts. Conversely, female never-smokers had the lower mortality risks across all outcomes, with HRs of 0.67 (0.64–0.70) for all-cause mortality and 0.42 (0.37–0.48) for CVD mortality, compared to male never smoker.

## Discussion

The results of this study reinforce the well-established association between tobacco smoking and increased mortality risk [23–26]. Current smokers exhibited significantly higher HRs for all-cause mortality, cardiovascular mortality, and cancer mortality compared to never-smokers. Smoking remains one of the most significant preventable risk factors for premature death worldwide, as it contributes to multiple fatal diseases, including CVD and cancer [1].

To address the potential sex differences in the association between tobacco smoking and mortality, two complementary analytical approaches were employed. First, we estimated sex-specific HRs by fitting separate Cox proportional hazards models for males and females, adjusting for the same set of covariates, allows us to describe and compare the magnitude of associations within the same sex group. Second, we formally tested for effect modification by sex through the inclusion of multiplicative interaction terms (sex × smoking) in fully adjusted models conducted in the total population.

Regarding sex differences, these analyses suggest that overall survival was higher among females across smoking categories, consistent with the lower baseline mortality observed in females in most populations. It is noteworthy that the HRs observed in men and women were largely comparable across smoking categories. Formal interaction analyses indicated that the association with all-cause and cardiovascular mortality may be somewhat stronger in females, although the effect sizes were modest and CIs overlapped. For cancer mortality, no sex interaction was observed. Taken together, these findings support the interpretation that smoking has detrimental effects on both sexes, with only limited evidence for differential vulnerability.

Furthermore, no significant interaction was found for cancer mortality, suggesting a comparable relative risk within the same sex group. Even low levels of cigarette consumption were associated with an increased risk of death, emphasizing that there is no safe level of tobacco exposure. Additionally, secondhand smoke exposure remains a major concern, further increasing mortality risks among non-smokers.

The toxic composition of cigarette smoke plays a fundamental role in its harmful effects, containing over 7000 chemical compounds, including numerous carcinogens and toxicants that promote oxidative stress, inflammation, endothelial dysfunction, and DNA damage [2]. These mechanisms contribute to the accelerated progression of cardiovascular and neoplastic diseases, ultimately leading to higher mortality rates among smokers.

The literature on sex-specific effects of smoking remains heterogeneous. Some studies suggest heightened susceptibility among women, possibly related to differences in airway size, hormonal metabolism of carcinogens, or cardiovascular risk factor profiles [27, 28], while others have reported comparable risks between sexes. Given this inconsistency, it is important to interpret our results with caution and avoid overemphasizing differences that the data do not strongly support. Rather, our findings reinforce that smoking cessation and prevention are critical priorities across both sexes, and that any potential sex-related nuances warrant further investigation through pooled analyses and mechanistic studies.

Although the greatest risk was observed among current smokers, past smoking remained significantly associated with an increased risk of all-cause, CVD, and cancer mortality compared to never-smokers [29]. These findings emphasize the long-term detrimental effects of tobacco on health, even after smoking cessation. However, the risk was lower in past smokers than in current smokers, reaffirming the substantial benefits of quitting smoking at any stage.

The persistence of excess mortality risk among former smokers may be attributed to irreversible damage caused by prolonged tobacco exposure [30]. For example, structural and functional impairments in the cardiovascular system, accumulated DNA mutations leading to carcinogenesis, and chronic lung injury may not fully recover even after smoking cessation [31]. However, the risk declines over time, suggesting that early cessation is crucial to reducing mortality. These results emphasize the importance of promoting early smoking prevention and sustained cessation efforts, regardless of sex. Healthcare providers should continue to emphasize the benefits of quitting and offer comprehensive support to individuals attempting to quit smoking.

These findings reaffirm the overwhelming contribution of tobacco smoking to premature mortality [32]. Participants with higher cumulative tobacco exposure, measured in pack-years, exhibited greater HRs for all-cause, CVD, and cancer mortality. This cumulative effect was observed for both males and females. This trend reinforces the notion that the harmful effects of smoking are cumulative, with prolonged exposure leading to an increasing burden of disease [33]. The mechanisms underlying this relationship include progressive vascular damage, chronic inflammation, and the accumulation of genetic mutations over time. These processes contribute to the development and progression of fatal diseases, particularly CVD and cancer [2].

Overall, our findings reaffirm the overwhelming contribution of tobacco smoking to premature mortality. A clear dose–response relationship was observed, with higher smoking intensity and cumulative exposure associated with progressively greater risks of all-cause, cardiovascular, and cancer mortality. Importantly, although former smokers continued to show an excess risk compared with never-smokers, their risks were consistently lower than those of current smokers, underscoring both the persistent harm of prior exposure and the substantial benefits of cessation. Early prevention of smoking initiation therefore remains the most effective strategy to reduce the lifetime burden of tobacco-related disease.

When considering sex-specific effects, the HRs observed in men and women were largely comparable across smoking categories. Although formal interaction analyses suggested slightly stronger associations for cardiovascular mortality in women, the effect sizes were modest, and CIs overlapped. For cancer mortality, no significant sex interaction was observed. These results indicate that the harmful impact of smoking is fundamentally similar in both sexes, with absolute differences primarily reflecting the lower baseline mortality risk among women.

Taken together, these findings highlight three key points: (i) smoking is a major driver of mortality with a clear dose–response relationship; (ii) cessation provides substantial health benefits, though some excess risk persists, emphasizing the importance of early prevention; and (iii) sex-related differences, while biologically plausible, appear minor in our cohort and should not detract from the overarching priority of universal smoking prevention and cessation policies.

### Strengths and limitations

This study’s primary strength lies in the expansive sample size provided by the UK Biobank cohort. A notable concern is the low response rate of 5.5% in the UK Biobank study, which could introduce participant bias. Despite this, the robust sample size and high internal validity make it unlikely that these limitations significantly influenced the observed associations. The study’s focus on middle-aged participants from the UK also limits the generalizability of its findings to other age groups and ethnicities. Nonetheless, the use of standardized protocols in data collection by the UK Biobank enhances the external validity of our results, ensuring consistent data collection across various conditions and personnel.

The study, however, is not without its limitations. One limitation is that our cause-specific Cox models did not formally account for competing risks. Deaths from causes other than the outcome of interest (e.g. non-CVD causes in the CVD model) were treated as censored. While this approach allows estimation of relative risks, future work could apply competing risk regression to further refine absolute risk estimations. Socioeconomic data, medical history, and comorbidities were primarily gathered through self-reported questionnaires or assessments during medical examinations at health centers. Additionally, the data collection period (2006–2010) may not accurately reflect current patterns and risks associated with tobacco use. Tobacco use was self-reported, rather than verified through urine or blood tests. No information during the follow-up was collected for tobacco use, thus, some participants may continue as long-term users or not. Consequently, it’s possible that the impact of prolonged, regular tobacco use was not fully captured in these findings. Future research, employing more precise measurements of tobacco use over extended periods, is necessary to validate these results. Additionally, as the precise dosage of tobacco was not consistently recorded across all study phases, the investigation was unable to determine if there is a dose-response relationship between tobacco use and mortality. This aspect presents an important way for exploration in upcoming studies.

## Conclusion

This study confirms the strong and graded association between tobacco smoking and premature mortality in a large UK population sample. While women exhibited lower absolute mortality than men, the relative risks linked to smoking were broadly comparable across sexes, indicating that tobacco remains uniformly harmful. These findings underscore the urgent need for universal prevention and cessation strategies, with early intervention as the most effective way to reduce the long-term burden of smoking-related disease.

## Supplementary Material

ckaf194_Supplementary_Data

## Data Availability

Alexandre Vallée had full access to all the data in the study and takes responsibility for the integrity of the data and the accuracy of the data analysis. Key points Tobacco smoking is a major risk factor for mortality, with strong associations observed for all-cause, cardiovascular, and cancer mortality. Based on sex-stratified models and interaction terms, relative risks were largely similar in males and females, with only modest indications of stronger associations for cardiovascular mortality in females. In absolute terms, mortality remains lower in women across smoking categories. A dose-response relationship was observed, where higher smoking intensity and cumulative exposure (pack-years) significantly increased mortality risk. Even past smokers had an elevated mortality risk, emphasizing the long-term health consequences of smoking.
