# Supplementary material for: Associations between tobacco smoking and mortality: a sex-stratified cohort analysis
Source: Eur J Public Health. 2025 Oct 24;35(6):1212–8. doi: 10.1093/eurpub/ckaf194 (PMC12707473; doi:10.1093/eurpub/ckaf194)

Supplementary Figure 1: Kaplan Meier for overall mortality (A), CVD mortality (B) and cancer mortality (C) in males and females according to smoke pack years classification


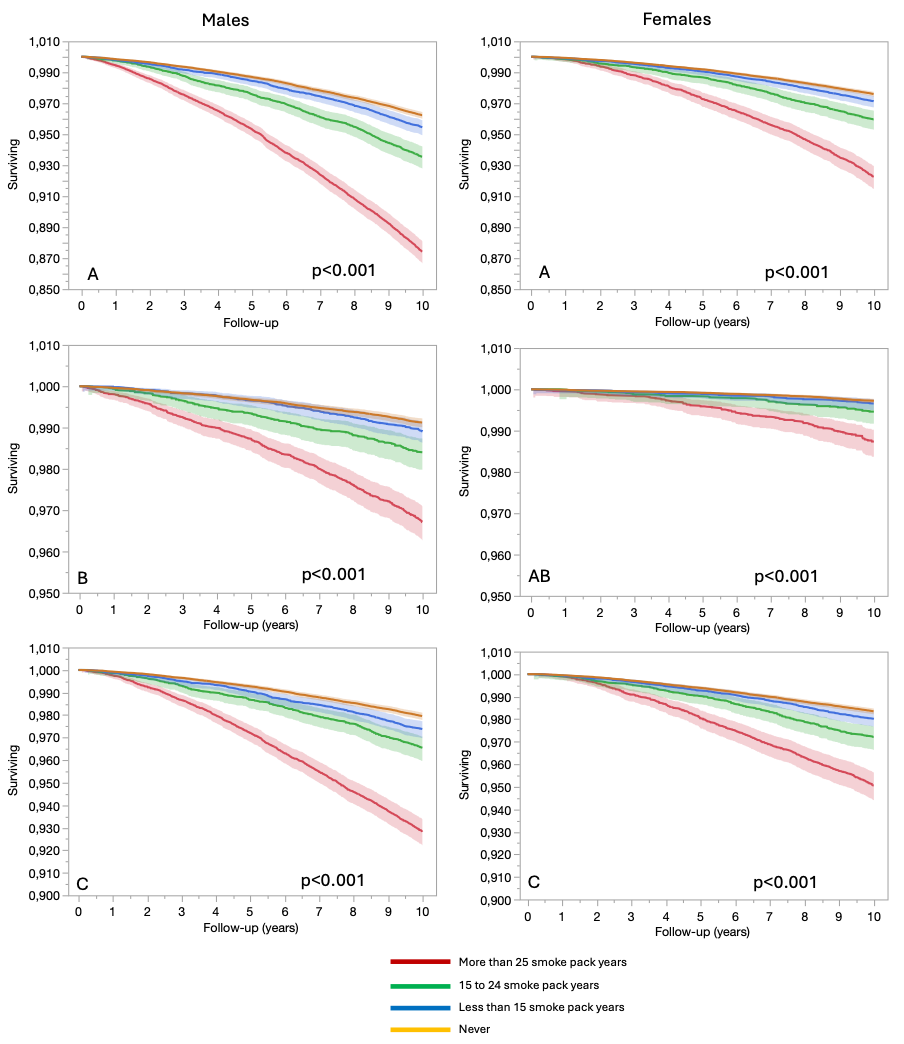

Supplement: ckaf194_Supplementary_Data [file ckaf194_supplementary_data.zip › ejph-2025-02-om-0152-File004.docx]
