# Supplementary material for: Associations between tobacco smoking and mortality: a sex-stratified cohort analysis
Source: Eur J Public Health. 2025 Oct 24;35(6):1212–8. doi: 10.1093/eurpub/ckaf194 (PMC12707473; doi:10.1093/eurpub/ckaf194)
Supplement: ckaf194_Supplementary_Data [file ckaf194_supplementary_data.zip › ejph-2025-02-om-0152-File006.docx]

**Supplementary Table 1:** Adjusted hazard ratios (HRs, 95% CI) for mortality according to combined sex-smoking groups (reference: male never smokers).

| **Sex-smoking group** | **HR mortality (IC95%)** | **HR CVD mortality (IC95%)** | **HR cancer mortality (IC95%)** |
| --- | --- | --- | --- |
| Male never smoker | Ref. | Ref. | Ref. |
| Male past smoker | 1.29 [1.25 – 1.35] | 1.45 [1.24 – 1.69] | 1.36 [1.29 – 1.44] |
| Male current smoker | 2.42 [2.31 – 2.55] | 2.62 [2.37 – 2.91] | 2.56 [2.38 – 2.74] |
| Female never smoker | 0.67 [0.64 – 0.70] | 0.42 [0.37 – 0.48] | 0.82 [0.77 – 0.87] |
| Female past smoker | 0.84 [0.79 – 0.88] | 0.54 [0.48 – 0.61] | 1.04 [0.97 – 1.11] |
| Female current smoker | 1.72 [1.61 – 1.84] | 1.17 [1.01 – 1.36] | 2.00 [1.83 – 2.18] |
